# The Immune Regulatory Role of Adenosine in the Tumor Microenvironment

**DOI:** 10.3390/ijms241914928

**Published:** 2023-10-05

**Authors:** Jianlei Xing, Jinhua Zhang, Jinyan Wang

**Affiliations:** 1 Department of Immunology, School of Basic Medicine, China Medical University, Shenyang 100001, China; 2 College of Life Science and Bioengineering, Beijing Jiaotong University, Beijing 100044, China

**Keywords:** adenosine, CD73, CD39, tumor immunotherapy

## Abstract

Adenosine, an immunosuppressive metabolite, is produced by adenosine triphosphate (ATP) released from dying or stressed cells and is found at high levels in the tumor microenvironment of most solid tumors. It mediates pro-tumor activities by inducing tumor cell proliferation, migration or invasion, tumor tissue angiogenesis, and chemoresistance. In addition, adenosine plays an important role in regulating anti-tumor immune responses and facilitating tumor immune escape. Adenosine receptors are broadly expressed by tumor-infiltrated immune cells, including suppressive tumor-associated macrophages and CD4^+^ regulatory T cells, as well as effector CD4^+^ T cells and CD8^+^ cytotoxic T lymphocytes. Therefore, adenosine is indispensable in down-regulating anti-tumor immune responses in the tumor microenvironment and contributes to tumor progression. This review describes the current progress on the role of adenosine/adenosine receptor pathway in regulating the tumor-infiltrating immune cells that contribute to tumor immune evasion and aims to provide insights into adenosine-targeted tumor immunotherapy.

## 1. Introduction

**Figure 1:**
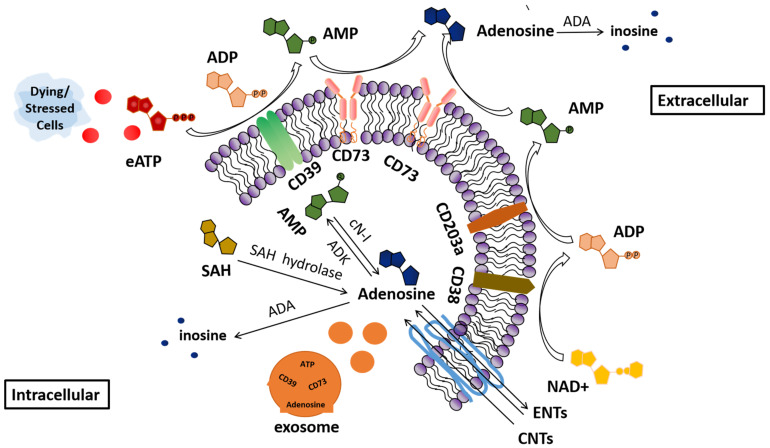
Adenosine production and degradation. Adenosine is mainly formed by consecutive extracellular adenosine triphosphate (eATP) dephosphorylation catalyzed by CD39 and CD73. NAD^+^ is released by the salvage pathway and hydrolyzed by CD38 to form ADP ribose. This is further degraded to AMP through CD203a. Following this, CD73 dephosphorylates AMP to adenosine. Intracellular adenosine can be produced by hydrolyzing AMP through cytoplasmic 5′-nucleotidase-I (cN-I) or by hydrolyzing S-adenosine homocysteine (SAH) through SAH hydrolase. The bioavailability of adenosine depends on its conversion to inosine via adenosine deaminase (ADA), which comes in both intracellular and extracellular forms, and adenosine could be transported by ENTs (both directions) and CNTs (one-way transport). Once inside the cell, adenosine is phosphorylated to AMP by adenosine kinase (ADK) or degraded to inosine by ADA. In addition, tumor-derived exosomes (TEX) could carry CD39, CD73 molecules and adenosine secreted outside the cell. eATP, extracellular adenosine triphosphate; AMP, adenosine monophosphate; ADP, adenosine diphosphate; cN-I, cytoplasmic 5′-nucleotidase-I; SAH, S-adenosine homocysteine; ADA, adenosine deaminase; ADK, adenosine kinase; NAD^+^, nicotinamide adenine dinucleotide; ENTs, equilibrative nucleoside transporters; CNTs, concentrative nucleoside transporters; P, phosphate group (the different colors are used to distinguish between ATP, AMP, ADP, and adenosine).

Adenosine, an endogenous purine nucleoside, plays an important role in regulating immune responses. Adenosine is mainly formed by consecutive extracellular adenosine triphosphate (eATP) dephosphorylation catalyzed by ectonucleoside triphosphate di-phosphohydrolase 1 (CD39) and ecto-5′-nucleotidase (CD73), in which CD39 initially mediates dephosphorylation of ATP to adenosine diphosphate (ADP) and adenosine monophosphate (AMP), then CD73 converts AMP to adenosine. Adenosine is degraded into inosine by adenosine deaminase (ADA), which occurs both intracellularly and extracellularly. Although the CD39/CD73 pathway is still considered to be the major source of adenosine in the tumor microenvironment (TME) [1,2], additional ectoenzymes also contribute to the metabolism of extracellular nucleotides. For instance, nicotinamide adenine dinucleotide (NAD^+^) can also be released in the hypoxic TME by the salvage pathway and can be hydrolyzed by CD38 to form ADP ribose. ADP is then further degraded to AMP through the CD38/CD203a/CD73 pathway [3,4,5]. Adenosine could be transported into the cell by ENTs (both directions) and CNTs (one-way transport). Intracellular adenosine can be produced by hydrolyzing AMP through cytoplasmic 5′-nucleotidase-I (cN-I) [6,7] or by hydrolyzing S-adenosine homocysteine (SAH) through SAH hydrolase. The generated adenosine is either phosphorylated to AMP by adenosine kinase (ADK) or degraded to inosine by ADA [8,9,10,11]. eATP is released by dying or stressed cells [12]. Meanwhile, tumor-derived exosomes (TEX) were shown to be associated with cellular stress, such as hypoxia, acidic pH, and many other triggers present in the TME [13]. Studies have shown that the CD39 and CD73 carried by TEX are enzymatically active and can produce adenosine [14,15] (Figure 1).

Under physiological conditions, the main role of adenosine is tissue protection and anti-injury to counteract the pro-immunogenic and pro-inflammatory activities of eATP [7]. However, under pathological conditions, increased levels of adenosine are involved in anti-inflammatory responses in the tissues and suppressive anti-tumor immunity in various cancers [16,17,18]. For example, extracellular adenosine concentrations are 10 to 20 times higher than normal levels in inflamed tissues in the context of ischemia, trauma, and inflammation [19,20,21,22]. Accumulating evidence shows that adenosine can also be produced at high levels as a metabolite in the TME of major solid tumors [23,24], serving as immune suppressive molecules that contribute to tumor immune escape by modulating various immune cells via receptor-dependent/receptor-independent mechanisms. Secondly, adenosine indirectly affects the concentration of other factors in the adenosinergic pathway, such as ATP, AMP [6], inosine [25], adenosine deaminase (ADAR) [26], and AMP-activated protein kinase (AMPK) [27]. These factors regulate the communication between tumor cells and immune cells by different mechanisms.

To restore immunosurveillance, largely by blocking adenosine-producing enzymes in the TME and adenosine receptors on immune cells [17]. There are four adenosine receptor subtypes, which belong to the family of G protein-coupled receptors (GPCRs), including A_1_, A_2A_, A_2B_, and A_3_ receptors (A_1_R, A_2A_R, A_2B_R, and A_3_R) [19]. A_1_R is mainly expressed in the central nervous system, whereas A_2B_R and A_3_R are mostly expressed in the peripheral tissues and participate in inflammation and immune responses. A_2A_R is predominantly expressed in various immune cells, both in central and peripheral lymphoid tissues. When adenosine levels are low, its interaction occurs preferentially with the A_1_R and/or A_3_R, activating the G_i/o_ protein and thus reducing adenylate cyclase (AC) and protein kinase A (PKA). At higher levels, adenosine activates the A_2A_R and/or A_2B_R components, activating the AC/cyclic AMP (cAMP)/PKA cascade through the G_s_ protein, thereby regulating intracellular cAMP levels that control the activity of various cells [28,29,30]. It has been demonstrated that A_2A_R is broadly expressed on several immune cells, including tumor-associated macrophages (TAMs), CD4^+^ regulatory T cells (Tregs), effector CD4^+^ T cells, and cytotoxic T lymphocytes at distant levels, in the TME of major solid cancers [8,31]. Therefore, adenosine may act prominently as a key regulator to control anti-tumor immunity and may serve as a potential target for tumor immunotherapy. Here, we describe the current progress on the role of the adenosine/adenosine receptor pathway in regulating the tumor-infiltrated immune cells that contribute to tumor immune escape and introduce the current status of targeted adenosine/adenosine receptor therapy. In addition, we evaluate the limitations of targeting this pathway, aiming to provide insights into adenosine-targeted tumor immunotherapy.

## 2. The Role of Adenosine on the Regulation of Various Immune Cells in the TME

Various immune cells infiltrate tumor tissues that control tumor progression. Some of these cells mediate tumor-antagonizing immune responses that suppress tumor progression, such as natural killer (NK) cells, dendritic cells (DCs), effector CD4^+^ T cells, and CD8^+^ T cells. Several immune cells may serve as suppressors that inhibit anti-tumor immunity, facilitating tumor progression. These cells include Tregs, TAMs, and myeloid-derived suppressor cells (MDSCs) [32,33]. Accumulating evidence shows that most of these cells express CD39/CD73 and/or adenosine receptors in the TME that could be redundantly regulated by adenosine, thus controlling tumor development [1,34,35,36].

### 2.1. Natural Killer Cells

NK cells arise from hematopoietic stem cells originating in the bone marrow and further differentiate from the common lymphoid progenitor cell. The activation of NK cells is determined by the balance of activating and inhibitory signals on their surface upon interaction with cognate ligands in potential target cells. NK cells can also be activated by cytokines alone without target cell interaction, such as interferon (IFN)-γ [37]. It has been shown that adenosine has specific immunomodulatory effects on the maturation, migration, and effector functions of NK cells [38,39,40]. In addition to inhibiting the maturation of NK cells and limiting the accumulation of cytotoxic CD56dim subsets [40], adenosine also prevents the transport of NK cells to tumor sites by changing the chemokine environment [41] and inhibits the function of NK cell effector molecules against tumor targets [42]. Tumor-infiltrating NK cells upregulate CD73 expression, and the frequency of these CD73^+^ NK cells correlate with larger tumor sizes in breast cancer patients. CD73^+^ NK cells undergo transcriptional reprogramming and upregulate interleukin (IL)-10 production via STAT3 transcriptional activity, suppressing CD4^+^ T cell proliferation and IFN-γ production [43].

Notably, adenosine inhibits the cytotoxic effect of NK cells mainly through A_2A_R signaling and causes tumor immune escape in several solid tumors, such as MethA sarcoma and 3LL Lewis lung carcinoma, through cAMP-dependent signaling that mediates PKA engagement [44,45,46]. A clinical study showed that CD56^dim^CD16^−^ and CD56^bright^CD16^−^ NK cells represent the predominant NK cell subpopulations in acute myeloid leukemia (AML) and that CD39^+^/CD38^+^ cells cluster on CD56^bright^CD16^−^ NK cells. Combined targeting of CD39 or A_2A_R significantly augments the anti-TIGIT-mediated lysis of AML cells [38]. Meanwhile, blocking the activity of the CD73 enzyme shows increased NK cell killing of tumor cells and an obvious anti-tumor response [47]. In addition, A_2B_R antagonists rescue T and NK cell proliferation, increase IFN-γ and perforin production, and increase tumor-infiltrating lymphocyte infiltration into breast cancer spheroids [48]. Therefore, targeting inhibition of adenosine/adenosine receptors may enhance NK cell activities that positively regulate the anti-tumor immune response that inhibits tumor progression.

### 2.2. Dendritic Cells

DCs play an important role in the initiation of T cell-mediated anti-tumor immune responses. While immature DCs exhibit a potent capacity for taken-up antigen and antigen processing, mature DCs show efficient presentation in the context of MHC molecules to T cells, thus initiating anti-tumor immune responses. Diverse DC subsets have been identified in the tumor microenvironment and tumor-draining lymph nodes, including the CD103^+^ cDC1 subset, CD11b^+^ cDC2 subset, and B220^+^ plasmacytoid DCs [49]. Adenosine has been found to negatively regulate the antigen presentation process of DCs in the TME. A_2A_R and/or A_2B_R^+^ DCs showed decreased CD4^+^ T cell priming and anti-tumor immune responses in the TME [50,51]. Conversely, blocking A_2A_Rs deprives DCs of their contact with naïve conventional T cells, which leads to suppressed T cell priming and antigen-specific T cell responses. Indeed, in colorectal cancer, melanoma, and fibrosarcoma in mice, an A_2A_R antagonist enhanced the antigen presentation capacity of CD103^+^ DCs as well as increased T cell function [52]. Furthermore, it has been shown that blocking CD73 signaling in patients with colon adenocarcinoma synergistically enhances oxaliplatin (OXP)-induced ATP release, a hallmark of immunogenic cell death, which promotes DC maturation and immune cell infiltration [53].

Adenosine also regulates the differentiation and maturation of DCs in the TME. Upon binding to adenosine, DCs preferentially differentiate into a myeloid DC population through a mechanism driven by expressing high levels of tolerogenic factors, such as COX-2, IDO, IL-6, IL-8, IL-10, TGF-β, and VEGF, thereby favoring the activation of suppressive CD4^+^ Tregs [51,54]. Constructed nanoparticles assembling small molecular A_2A_R inhibitors showed enhanced DC activation and increased infiltrating CD8^+^ T cells in the TME, leading to suppression of tumor progression [55].

### 2.3. Effector CD4^+^ T Helper Cells

CD4^+^ T cells play an important role in tumor immunity. Upon activation in response to antigen stimulation in the context of MHC class II molecules, CD4^+^ T cells undergo proliferation and differentiation to generate effector CD4^+^ T cells in the draining lymphoid tissues of the tumor. CD4^+^ T cells secrete different cytokine profiles that are closely associated with anti-tumor immunity in various cancers. T helper 1 (Th1), T helper 2 (Th2), and T helper 17 (Th17) cells have been found in the TME of various inflamed cancers. It is believed that Th1 cells promote CD8^+^ T cell immunity to tumor cells as well as enhance anti-tumor immune responses by recruiting inflammatory cells, such as macrophages, granulocytes, and NK cells, to the site of tumor tissue. In contrast, Th2 cells are required for priming B cell activation and anti-tumor antibody production [56,57]. Studies have shown that adenosine inhibits the proliferation of Th1 and Th2 cells through binding to A_2A_R on the surface of cells [58,59]. The role of adenosine in the differentiation and function of Th17 cells remains unclear. Some studies showed that both CD73^+^ Th17 cells and CD39^+^ Th19 cells may function as immune suppressor cells instead of effector cells, with increased IL-10 production that favors the development of cancer in various murine models, including EL4 thymoma, B16F10 melanoma, LLC lung carcinoma, and MC38 colon carcinoma. Furthermore, infiltrated CD39^+^ Th17 cells in the TME are proportional to poor clinical outcomes in cancer patients [60,61]. Interestingly, adenosine and an A_2A_R agonist (PSB0777) promoted IL-17A and IL-8 production from human peripheral blood mononuclear cells in response to *Candida albicans* stimulation, suggesting a role for the adenosine/A_2A_R pathway in Th17 cell differentiation [62]. In addition, in vivo experiments in mice showed that A_2B_R promotes Th17 differentiation by augmenting IL-6 production by DCs, independent of intracellular cAMP, suggesting a different mechanism for Th17 cell differentiation by A_2B_R [63].

### 2.4. Cytotoxic CD8^+^ T Cells

CD8^+^ T cells are the most potent killing cells, with the ability to specifically recognize and eradicate immunogenic cancer cells. However, tumor-reactive CD8^+^ T cells become dysfunctional in the context of suppressive TMEs with the development of tumor progression. Adenosine is the key suppressive molecule in the TME and may induce tumor-reactive CD8^+^ T cells to become dysfunctional. Studies have shown that CD8^+^ T central memory cells (TCM) in the TME express high levels of A_2A_R that are susceptible to regulation by adenosine, leading to CD8^+^ T cells being functionally exhausted in the TME [64,65]. In addition, memory CD8^+^ T cells highly expressed CD73, which contrasts with terminally differentiated effector cells that did not express CD73 [66]. Studies have shown that tumor-infiltrating CD39^+^CD8^+^ T cells show less cytotoxicity compared with those of CD39^−^CD8^+^ T cells, which are characterized by the production of IFN-γ, tumor necrosis factor (TNF)-α, and granzyme B, suggesting that tumor-infiltrating CD8^+^ T cells with high CD39 expression exhibited features of exhaustion [67]. Indeed, CD39^+^CD8^+^ T cells exhibited a CD69^+^PD-1^+^perforin^low^IFNγ^low^ “exhausted” phenotype [68].

In addition, CD73 may control glucose uptake by CD8^+^ T cells through the production of adenosine, resulting in a decrease in the efficiency of T cells to control tumor growth. CD73-deficient CD8^+^ T cells showed increased glucose uptake and mitochondrial respiration and improved anti-tumor ability in melanoma-bearing mice [69]. The expression level of CD73 on CD8^+^ T cells is regulated by costimulatory signals initiated by the binding of CD28 on the surface of CD8^+^ T cells to the B7 molecules on target cells. In the absence of CD28 costimulation, CD73 expression levels on CD8^+^ T cells are upregulated in the TME in several solid cancers [70].

### 2.5. Tumor-Associated Macrophages

TAMs play an important role in regulating tumor progression and chemotherapy resistance. Macrophages display highly tumor environment-dependent plasticity that varies their biological function. Macrophages in the TME can be polarized into opposite functional states, known as M1 and M2 polarization [71,72,73]. M1 and M2 are classified as highly simplified models of complex functional behavior and cellular plasticity. The M1 phenotype is characterized by the expression of high levels of proinflammatory cytokines, high production of reactive nitrogen and oxygen intermediates, promotion of the Th1 response, and strong microbicidal and tumoricidal activity. In contrast, M2 macrophages are considered to be involved in parasite containment, promotion of tissue remodeling, and tumor progression and have immunoregulatory functions [74,75,76]. Many studies have shown that adenosine is a necessary element for tumor-induced macrophage proliferation. Macrophages secrete GM-CSF and enhance the expression of A_2A_R on macrophages in the presence of adenosine, thus initiating macrophage proliferation in hepatocellular carcinoma (HCC). Mechanistic analysis showed that tumor-derived adenosine binds to A_2A_R of TAMs, promoting M2-like macrophage polarization as well as proliferation via the activation of phosphatidylinositol-3-kinase (PI3K)/Akt and MEK/ERK pathways [77].

Previous studies have shown that the PI3Kγ signaling pathway of TAMs inhibits the activation of NF-κB through Akt and mTOR, which mediates immunosuppression to promote tumor growth. At the same time, the PI3Kγ signal in TAMs inhibits the recruitment of CD8^+^ T cells around the tumor [78,79]. High expression of A_2A_Rs on tumor cells promotes the secretion of chemokines and polarizing factors by activating the PI3K/AKT/NF-κB pathway, thereby promoting the migration and invasion of TAMs [80]. Although the main population of TAMs is immunosuppressive M2 macrophages, TAMs can be reprogrammed into M1 helper macrophages [78,79]. PI3Kγ inhibition can reverse these effects of TAMs by polarizing macrophages into NF-κB-dependent pro-inflammatory M1 macrophages [78]. Therefore, it is of great significance to identify the key checkpoint where TAMs are reprogrammed into M1 macrophages to further promote the killing effect of M1 macrophages on tumors. A_2A_R knockdown increases M1 polarization in TAMs [80,81]. In ovarian cancer, inhibition of CD39 or CD73 can reverse the suppression of T cell proliferation mediated by TAMs [82]. Hypoxia can induce the expression of A_2A_R and A_2B_R, and reduce the expression of adenosine kinase and balanced nucleoside transporters. In addition, the hypoxia microenvironment and tumor mTOR signal can stabilize or induce the expression of HIF-1α, respectively, and HIF-1α can induce the expression of CD39 and CD73 [75]. Therefore, metabolic changes in the TME promote the accumulation of adenosine in the interstitial space, and hypoxia further enhances the level of adenosine in the TME, which leads to immunosuppression by promoting M2 polarization. In addition, CD73 antibody triggers a strong accumulation of M1-type macrophages in non-small-cell lung cancer [83]. The metabolic changes of adenosine are very important for the reprogramming of TAMs. Blocking the adenosine/adenosine receptor pathway to promote M1 polarization of TAMs to establish an effective anti-tumor response is expected to become another auxiliary means of anti-tumor immunotherapy.

### 2.6. Myeloid-Derived Suppressor Cells

Bone marrow myeloid-derived suppressor cells (MDSCs) are a heterogeneous immune suppressive population that can be granulocytic or monocytic. MDSCs may mediate immune suppression via multiple mechanisms, including the release of proangiogenic factor VEGF, matrix remodeling enzyme MMPs, and inhibitory cytokine TGF-β [84]. It has been shown that MDSCs produce extracellular adenosine by expressing CD39 and CD73 [85,86]. Genetic ablation of CD73 led to decreased MDSCs in orthotopic mouse models of pancreatic ductal adenocarcinoma (PDAC) [87]. The high expression of adenosine produced by MDSCs in the TME may promote the proliferation of MDSCs themselves and their immunosuppressive activity in the mouse model of Lewis lung cancer [85]. Adenosine also improves the survival of MDSCs in the TME. Hypoxia is the characteristic feature of HCC that contributes to tumor progression. Hypoxia induces enhanced expression of CD39 through stabilization of HIF-1 in the tumor tissue of HCC, thereby preventing differentiation but promoting the survival of MDSCs [88].

A study showed that CD73-expressing MDSCs in the TME exhibited superior T cell suppressor function compared with CD73^−^ MDSCs in murine tumor models, including lung, colon, and melanoma. Mechanistically, tumor-derived prostaglandin E2 (PGE2), induces CD73 expression in MDSCs via both the STAT3 and CREB signaling pathways [89]. MDSCs express both A_2A_R and A_2B_R on their surface and are thus regulated by adenosine in an autocrine manner. It has been demonstrated that an A_2A_R inhibitor reduced the accumulation of MDSCs in the TME, improved DC activation, and increased CD8^+^ T lymphocyte infiltration [55]. Administration of an A_2B_R agonist had increased tumor growth in melanoma-bearing mice that was associated with increased accumulation of CD11b^+^Gr1^+^ MDSCs in the TME as well as higher levels of IL-10 and MCP-1. Conversely, the pharmacological blockade of A_2B_R reversed this suppressive effect in the TME, leading to a significant melanoma growth delay [90,91].

### 2.7. CD4^+^ Regulatory T Cells

CD4^+^ Tregs function as immune suppressor cells that negatively regulate anti-tumor immunity in most cancers. Tregs regulate anti-tumor immune responses through multiple mechanisms, including the production of adenosine through co-expression of CD39/CD73, granzyme B, perforin, or Fas/FasL pathways, and the development of tolerogenic DCs, leading to the formation of a regulatory T cell subset in the TME [92,93]. Studies have shown that tumor-infiltrating Tregs with higher expression of CD39 and CD73 displayed stronger immunosuppressive function compared with those in the draining lymph nodes of tumor-bearing mice [1,94]. CD39^+^CD73^+^ Tregs increase their proliferation rate and immunosuppressive function in an autocrine manner [95,96]. Furthermore, adenosine-A_2A_R signaling also promotes the induction of Foxp3^+^Treg cells from the CD4^+^Foxp3^−^ T cells that suppress effector T cell-mediated anti-tumor responses, promoting tumor progression [21,97]. In addition, tumor-derived adenosine also recruits CD4^+^ Treg cells to the TME, causing an immunosuppressive microenvironment. Tang et al. showed that binding tumor cell-derived adenosine to A_2A_R enhances CD73 transcription and upregulates chemokine CCL5 through activation of the p38/STAT1 pathway, recruiting Tregs to pancreatic tumors [97]. Adenosine produced by CD4^+^ Tregs suppresses the effector function of CD8^+^ and CD4^+^ T cells upon binding to corresponding A_2A_R on the effector cells. Another study showed that CD4^+^ Treg cells suppress the immune response through the increase of cAMP in target cells by the expression of COX-2 and the production of PGE2. The mechanisms responsible for Treg-mediated suppression involve the binding of adenosine and PGE2 produced by Tregs to their respective receptors expressed on T effector cells, leading to the up-regulation of adenylate cyclase and cAMP activities in T effector cells and to their functional inhibition [98,99]. These studies indicate that the presence of adenosine and PGE2 in the TME may synergistically mediate a powerful suppression of anti-tumor immunity, resulting in the progressive development of tumors.

**Figure 2:**
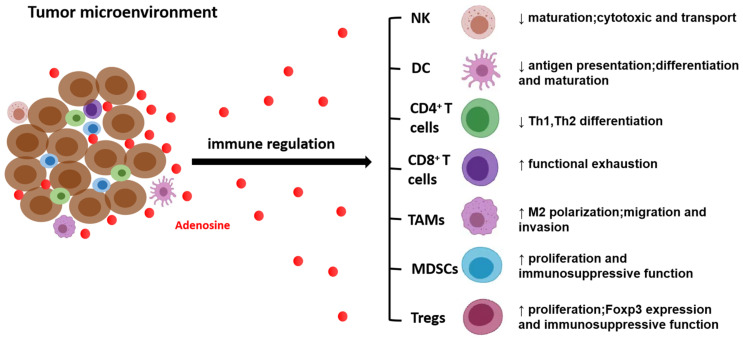
Adenosine plays an important role in tumor immunosuppression by interacting with different receptors of different immune cells. ↑, upregulate; ↓, downregulate; →, adenosine acts on different immune cells.

Type 1 regulatory (Tr1) T cells are Foxp3^−^ regulatory T cells, which are characterized by the predominant production of IL-10 and the expression of inhibitory receptors, such as LAG3 and CD49b, on the surface [100,101]. CD39^+^CD73^+^ Tr1 cells are found in the tumor microenvironment that facilitates the production of adenosine by hydrolyzing exogenous ATP/ADP [102]. A study showed that CD39^+^CD73^+^ Tr1 cells produce both adenosine and PGE2 in the TME, which promotes the development of head and neck squamous cell carcinoma. The inhibitory function of Tr1 was blocked by the usage of A_2A_R or EP2R antagonists (inhibitors of the PGE2 pathway), which confirmed that both adenosine and PGE2 were involved in Tr1-mediated immunosuppression in several solid tumors [99,103] (Figure 2).

## 3. Adenosine/Adenosine Receptor Blockade

### 3.1. Targeting CD39 or CD73

Due to the prominent suppressive role of adenosine in the TME that contributes to tumor development and progression, targeting adenosine pathways shows great promise in tumor immunotherapy [104,105,106]. Studies showed that inhibiting CD39 on macrophages in glioblastoma significantly increases their production of TNF and IL-12 while decreasing IL-10 secretion [107,108]. Meanwhile, in mouse melanoma and colorectal tumor models, TAMs and endothelial cells with high CD39 expression were effectively depleted following anti-CD39 treatment, thereby blocking angiogenesis [109]. CD39-specific antibodies suppressed the expression of CD39 mRNA and protein in murine colon adenocarcinoma, human breast cancer, and primary human T cells. This improved CD8^+^ T cell proliferation and substantially reduced the frequency of intratumoral Tregs [110].

The study on the mechanism of the anti-CD39 monoclonal antibody by Li et al. shows that anti-CD39 may bind to CD39 on intratumoral macrophages and monocytes expressing the P2X7 receptor, causing the release of eATP and triggering the activation of NALP3 inflammatory bodies. The downstream activation and release of IL-18 and IL-1β may promote the proliferation of CD8^+^ T cells and the effect function mediated by IFN-γ. It was revealed that the eATP-P2X7-ASC-NALP3 inflammatory body-IL18 pathway plays an important role in blocking the anti-tumor activity mediated by the CD39 enzyme, rather than simply reducing adenosine as the mechanism [1]. ATP degradation mediated by CD39 and CD73 eliminates the ability of apoptotic cells to recruit monocytes. ATP can induce a change in cell membrane permeability, resulting in the flow of Na^+^ and Ca^+^ into cells, which may result in growth inhibition [2]. Silva et al. demonstrated that activation of P2RX7 by eATP can promote metabolic adaptation and survival of the most persistent and functionally related memory CD8^+^ T cell population [111]. Drug blocking of CD39 prevented the degradation of eATP and enhanced macrophage phagocytosis of antibody-coated lymphoma cells in a P2X7 receptor-dependent manner [112].

Studies have shown that high expression of CD73 is associated with tumor development and a poor prognosis [97,113,114]. In glioblastoma, CD73 blockade was found to induce tumor cell apoptosis. Meanwhile, the population of Tregs, microglia, and macrophages was significantly reduced in the tumor microenvironment, but IL-6, CCL17, and CCL22 increased [113]. It was shown that CD39 and CD73 expression were significantly associated with poor survival in human PDAC samples and that the favorable prognostic effect associated with the presence of tumor-infiltrating CD8^+^ T cells was abolished. Although inhibition of CD39 or CD73 alone significantly slowed tumor growth in vivo [115,116], targeting these two nucleotidases showed significantly better anti-tumor activity [117]. Therefore, blocking the adenosine pathway may have double immunosuppressive effects: one is to promote the anti-tumor activity of effector T, NK, and other cells by blocking the accumulation of adenosine in the TME, and the other is to inhibit the proliferation of tumor cells by increasing eATP and providing essential sensor molecules to attract antigen-presenting cells to the tumor site. Several therapies targeting CD39 or CD73 have entered clinical trials (Table 1).

Interestingly, an increasing number of studies have shown that CD39 molecules, but not CD73 molecules, are considered co-inhibitory receptor molecules [118,119]. The conversion of ATP to ADP/AMP in the TME is regulated by CD39, leading to an increase in the AMP/ATP ratio and subsequent activation of AMPK [120]. AMPK serves as a central guardian for maintaining energy homeostasis by orchestrating diverse cellular processes, such as lipogenesis [121], glycolysis [122], the tricarboxylic acid cycle (TCA cycle) [123], cell cycle progression [124], and mitochondrial dynamics [125]. At the start, AMPK serves as a suppressor of tumors, potentially by working against the metabolic and signaling shifts that arise in cancer cells, such as heightened lipogenesis and the activation of mTORC1. In the event that tumorigenesis does take place, however, AMPK transitions to promoting the growth of the tumor, as it shields the tumor cells from the stresses that arise from their speedy proliferation [126]. A study has shown that in mouse models of colon cancer and fibrosarcoma, AMPK promotes lipid peroxidation by mediating phosphorylation of BECN1 and leads to ferroptosis of tumor cells [127]. In addition, in mouse melanomas, AMPK activates p38 MAPK, which inhibits PD-1 expression in Tregs by phosphorylating GSK-3β [128]. However, Cai Z et al. demonstrated that AMPK, activated in mouse metastasis models, drives pyruvate dehydrogenase complex (PDHc) activation to maintain the TCA cycle and promote breast cancer metastasis by adapting cancer cells to metabolic and oxidative stresses [123]. It is well documented that AMPK possesses double-edged sword characteristics in the context of tumor development and progression by modulating the inflammatory and metabolic pathways [129].

Otherwise, the CD39 molecule can also influence the level of eATP in the TME. Continuously, eATP continuously regulates the polarization of macrophages and the antigen presentation of DCs [1,119]. Therefore, the significant effects of CD39 may be due not only to its role as a rate-limiting enzyme in the adenosinergic pathway [19,119], but also to its indirect regulation of eATP or AMPK in the TME.

### 3.2. Targeting A_2A_R or A_2B_R

Adenosine plays a pro-tumor role in the TME mainly by interacting with A_2A_R on immune cells. Therefore, an increasing number of studies have focused on targeting A_2A_R. In the mouse model of chronic lymphoblastic leukemia, targeting A_2A_R had no effect on the size and weight of the tumor but saved the dysfunction of immune cells by reducing the accumulation of Tregs, restoring the expression of CD107a on T cells, and increasing the secretion of IL-2 and IFN-γ, indicating that anti-A_2A_R affects the function of immune cells rather than tumor cells [130,131,132]. Studies showed that targeting A_2A_R may enhance T cell activation and effector function in several murine cancer models, including MC38, CT26, RENCA, and B16. Notably, anti-A_2A_R could induce systemic anti-tumor immunity and increase memory formation that prevents tumor recurrence [133]. However, Festag et al. showed that T cell proliferation was inhibited by adenosine not through binding to adenosine receptors, but by intracellular downstream metabolites of adenosine, further blocking the synthesis of DNA, thereby inhibiting T cell proliferation and promoting T cell apoptosis in human T cells [134]. This may indicate that blocking CD39 or CD73 is more effective than blocking adenosine receptors. Adenosine inhibits T cell proliferation, activation, and apoptosis in different ways, but whether CD39 or CD73 blockers are superior to adenosine receptor blockers remains to be further verified [131,135,136].

Of the two major adenosine receptors responsible for immunosuppressive activity in the TME, A_2B_R has received much less attention than the high-affinity A_2A_R. A_2A_R is mainly expressed on the surface of T cells and NK cells. In contrast, A_2B_R is a low-affinity receptor mainly expressed on myeloid cells, including DCs, macrophages, and MDSCs, as well as cancer-associated fibroblasts (CAFs) [137]. A study showed that high expression of A_2B_R leads to worse outcomes in lung cancer patients. Mechanistically, inhibition of A_2B_R increases the glycolysis of DCs and promotes metabolic reprogramming of DCs to a more immunogenic state [138]. In a melanoma model, blocking A_2B_R stimulated T cell-mediated immunosurveillance by impairing the influx of MDSCs into the TME [139]. Due to the high concentration of adenosine in the TME, A_2B_R has a role that cannot be ignored, so the combination of A_2A_R and A_2B_R may provide a more comprehensive effect. However, there are currently only a few therapies that single-target CD39 or CD73 in clinical trials (Table 2).

### 3.3. Targeting CD38

As a bypass pathway for adenosine production, CD38 is the main NAD-degrading enzyme in several mammalian tissues. Studies have shown that CD38 is highly and uniformly expressed at the cell surface of multiple myeloma (MM) cells, and monoclonal antibodies against CD38 are highly efficacious in the treatment of MM [140,141,142]. In non-small-cell lung cancer, CXCR4 inhibitors can reduce the expression of CD73, CD38, and IL-10 on metastasis-initiating cells, thereby rescuing the cytotoxic activity of T cells and preventing TAM polarization, possibly by causing a decrease in adenosine and IL-10 production. This can effectively control the migration or invasion of lung tumor cells in vitro [143]. Interestingly, in HCC, patients with high CD38^+^CD68^+^ macrophage density had a better median overall survival of 34.43 months compared with 9.66 months in patients with low CD38^+^CD68^+^ macrophage density. CD38hi macrophages produce more IFN-γ and related cytokines, which may explain their predictive value when treated with immune checkpoint inhibitors [144]. CD38 has also been identified as a cell surface marker in hematologic cancers such as MM, but the effects of CD38 on different immune cells and other cancers are still being explored [142]. More than 100 therapies targeting CD38 are being investigated clinically, of which eight have entered phase 3 clinical trials (Table 3).

### 3.4. Combination Therapy

Cancer immunotherapy has made great progress and shown better efficacy than conventional chemical therapies for several malignancies in these decades. Immunotherapy, including immune checkpoint inhibitors and adoptive cell therapy, has demonstrated objective clinical responses in several cancers. For example, monoclonal antibodies against PD-1 showed satisfactory clinical advantages and are recommended for first- or second-line treatments in some chemical drug-resistant cancers [145,146,147]. Anti-tumor efficacy was significantly enhanced when used in combination with other immunotherapies, including the recovery of immune responses in models with incomplete responses to anti-PD-L1 or anti-CTLA-4 monotherapy [148].

Several studies have shown that the combination of anti-CD39 and anti-PD1 is more effective in the treatment of tumors than a single treatment in the MC38 tumor model. Furthermore, anti-CD39 can transform anti-PD1-resistant tumors into sensitive tumors, thereby transforming “cold” tumors into “hot” tumors. Mechanistically, anti-CD39 increases the proliferation of tumor-infiltrating lymphocytes, while anti-PD-1 may restore the dysfunctional phenotype of these lymphocytes [1,119,149]. The combinational blockade of CD73 and PD-1 also showed promising tumor suppression. The immunomodulatory mechanism of CD73 blockers is different from that of PD-1 blockers in a mouse colorectal cancer model. Anti-CD73 enhanced the anticancer function of immunosuppressive Tregs and depleted T cells, while PD-1 blockers quantitatively decreased Malat1^high^ Treg and M2 macrophages. PD-1 blocking induces Treg deletion, and anti-CD73 therapy increases the activation of CD8^+^ T cells [137,150].

Anti-A_2A_R combined with either anti-PD-L1 or anti-CTLA-4 therapy showed efficacious tumor inhibition and led to up to 90% of tumors being eliminated in MC38 tumor-bearing mice that did not respond fully to anti-PD-L1 or anti-CTLA-4 monoclonal antibodies alone. Therefore, prioritizing strategies that offer combination therapy and target the adenosine pathway and immunotherapy in cancer is of great importance. Reportedly, more than 50 combination therapies targeting the ATP-adenosine pathway through CD73 or A_2A_R/A_2B_R antagonists are being explored clinically [151]. For example, several adenosine/adenosine receptor pathway blockers combined with PD-1 blockers have been used in clinical trials (Table 4).

## 4. Limitation and Prospect

**Figure 3:**
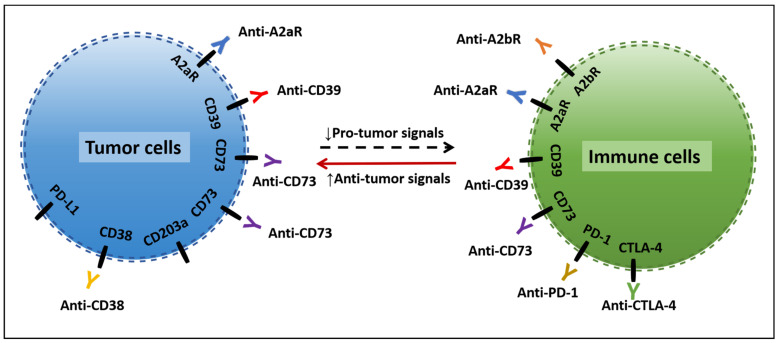
Targeting adenosine/adenosine receptor pathways in tumor and immune cells. Adenosine/adenosine receptor blocking works collaboratively with immune checkpoint blockers like PD-1 and CTLA-4 inhibitors to promote anti-tumor immunity and inhibit pro-tumor immune responses through different mechanisms. ↑, upregulate; ↓, downregulate; dotted arrow, pro-tumor signals; red arrow, anti-tumor signals; dashed lines around cells, the schematic structure of the cell membrane.

A variety of metabolic regulation methods of adenosine levels have been developed, and several clinical studies have been carried out to evaluate the initial efficacy of new inhibitors of CD39, CD73, or A_2a_R signaling pathways in cancer therapy. It’s worth noting that blocking the adenosine/adenosine receptor pathway combined with immune checkpoint inhibitors such as anti-PD-1 or anti-CTLA-4 can significantly enhance the efficacy of anti-PD-1 or anti-CTLA-4, including in anti-PD-1-/anti-PD-L1-tolerant tumor types. In summary, the adenosine/adenosine receptor pathway is expected to be another important target for tumor immunotherapy (Figure 3).

The high-affinity A_2A_R adenosine signaling pathway in tumor tissues is the mainstream research direction because it is considered to effectively inhibit the immune response in tumor and normal tissues. However, it must be considered that other adenosine receptors may be more important; as mentioned earlier, the affinities of the adenosine receptors are different, and the level of extracellular adenosine depends on tissue, treatment, and intensity in time and space. In addition, it is important to consider that, as a whole, the TME is composed of tumor cells, immune cells, stromal cells, and metabolites. The other metabolites in the adenosinergic pathway, such as ATP, inosine, and AMPK, should be fully considered. Therefore, clarifying the specific mechanism of each adenosinergic pathway-related factor and the results of its combined action will provide strong theoretical support for us to combine adenosine and its receptor pathways and metabolic regulation in the future.

## Figures and Tables

**Table 1:** Studies targeting CD39 or CD73 in cancer.

Study ID	Conditions	Interventions	Phase	Start Date
NCT04306900	Solid Tumor, Adult	COMBINATION_PRODUCT: TTX-030, budigalimab and mFOLFOX6|COMBINATION_PRODUCT: TTX-030, budigalimab and docetaxel|COMBINATION_PRODUCT: TTX-030 and mFOLFOX6|COMBINATION_PRODUCT: TTX-030 and budigalimab|COMBINATION_PRODUCT: TTX-030, budigalimab, nab-paclitaxel and gemcitabine|COMBINATION_PRODUCT: TTX-030 and pembrolizumab|COMBINATION_PRODUCT: TTX-030, nab-paclitaxel and gemcitabine|COMBINATION_PRODUCT: Budigalimab and mFOLFOX6	PHASE1	30 March 2020
NCT05374226	Advanced Solid Tumors or Lymphomas	BIOLOGICAL: JS019	PHASE1	31 March 2022
NCT05508373	Advanced Solid Tumors	BIOLOGICAL: JS019	PHASE1	29 March 2022
NCT05075564	Advanced Solid Tumor	DRUG: Part 1 ES002023|DRUG: Part 2 ES002023	PHASE1	23 December 2021
NCT05381935	Advanced Solid Tumor	DRUG: ES014|DRUG: ES014	PHASE1	21 April 2023
NCT04668300	Metastatic Angiosarcoma|Metastatic Dedifferentiated Liposarcoma|Metastatic Osteosarcoma|Recurrent Angiosarcoma|Recurrent Dedifferentiated Liposarcoma|Recurrent Osteosarcoma|Refractory Dedifferentiated Liposarcoma|Refractory Osteosarcoma	BIOLOGICAL: Durvalumab|BIOLOGICAL: Oleclumab	PHASE2	26 November 2020
NCT04148937	Advanced Cancer	DRUG: LY3475070|DRUG: Pembrolizumab	PHASE1	16 January 2020
NCT05174585	Solid Tumor	BIOLOGICAL: JAB-BX102 (anti-CD73 monoclonal antibody)|BIOLOGICAL: pembrolizumab (anti-PD-1 monoclonal antibody)	PHASE1|PHASE2	18 August 2022
NCT05329766	Gastrointestinal Tract Malignancies	DRUG: Domvanalimab|DRUG: Quemliclustat|DRUG: Zimberelimab|DRUG: Fluorouracil|DRUG: Leucovorin|DRUG: Oxaliplatin	PHASE2	10 June 2022
NCT04797468	Advanced Solid Tumor	DRUG: HLX23	PHASE1	18 July 2022
NCT05688215	Borderline Resectable Pancreatic Adenocarcinoma|Locally Advanced Pancreatic Ductal Adenocarcinoma	PROCEDURE: Biospecimen Collection|PROCEDURE: Computed Tomography|PROCEDURE: Core Biopsy|DRUG: Fluorouracil|DRUG: Irinotecan|DRUG: Leucovorin|DRUG: Leucovorin Calcium|DRUG: Oxaliplatin|DRUG: Quemliclustat|DRUG: Zimberelimab	PHASE1|PHASE2	7 March 2023
NCT04572152	Advanced or Metastatic Solid Tumors	BIOLOGICAL: AK119|BIOLOGICAL: AK104	PHASE1	18 January 2021
NCT03954704	Advanced Solid Tumors	DRUG: Dalutrafusp alfa|DRUG: mFOLFOX6 Regimen|DRUG: dalutrafusp alfa	PHASE1	3 June 2019
NCT04672434	Metastatic Cancer|Solid Tumor	DRUG: Sym021|DRUG: Sym024	PHASE1	19 November 2020
NCT03875573	Luminal B	DRUG: Durvalumab|RADIATION: Stereotactic Body Radiotherapy|DRUG: Oleclumab	PHASE2	6 November 2019
NCT03454451	Non-Small-Cell Lung Cancer|Renal Cell Cancer|Colorectal Cancer|Triple Negative Breast Cancer|Cervical Cancer|Ovarian Cancer|Pancreatic Cancer|Endometrial Cancer|Sarcoma|Squamous Cell Carcinoma of the Head and Neck|Bladder Cancer|Metastatic Castration Resistant Prostate Cancer|Non-hodgkin Lymphoma	DRUG: CPI-006|DRUG: CPI-006 + ciforadenant|DRUG: CPI-006 + pembrolizumab|DRUG: CPI-006|DRUG: CPI-006 + ciforadenant|DRUG: CPI-006 + pembrolizumab	PHASE1	25 April 2018
NCT05632328	Advanced Pancreatic Ductal Adenocarcinoma|Pancreatic Ductal Adenocarcinoma|Pancreatic Cancer	DRUG: AGEN1423|DRUG: Balstilimab|DRUG: Gemcitabine|DRUG: Nab-paclitaxel	PHASE2	23 April 2023
NCT05559541	Solid Tumor, Adult	DRUG: AK119|DRUG: AK104	PHASE1|PHASE2	15 December 2022
NCT05689853	Solid Tumor, Adult	DRUG: AK119|DRUG: AK112	PHASE1|PHASE2	14 April 2023
NCT04940286	Borderline Resectable Pancreatic Adenocarcinoma|Resectable Pancreatic Adenocarcinoma|Stage IA Pancreatic Cancer AJCC v8|Stage IB Pancreatic Cancer AJCC v8|Stage IIA Pancreatic Cancer AJCC v8|Stage IIB Pancreatic Cancer AJCC v8	BIOLOGICAL: Durvalumab|DRUG: Gemcitabine|DRUG: Nab-paclitaxel|BIOLOGICAL: Oleclumab	PHASE2	28 September 2021
NCT04660812	Metastatic Colorectal Cancer	DRUG: AB680|DRUG: Etrumadenant|DRUG: Zimberelimab|DRUG: Bevacizumab|DRUG: m-FOLFOX-6 regimen|DRUG: Regorafenib	PHASE1|PHASE2	10 May 2021
NCT05143970	Metastatic Cancer|Metastatic Breast Cancer|Metastatic Pancreatic Cancer|Metastatic Gastric Cancer|Metastatic Lung Cancer|Metastatic Ovary Cancer|Oesophageal Cancer|Endometrial Cancer|Advanced Solid Tumor	DRUG: IPH5301 ALONE OR IN COMBINATION WITH CHEMOTHERAPY AND TRASTUZUMAB	PHASE1	21 January 2022
NCT05431270	Metastatic Cancer|Refractory Cancer|Non-Small-Cell Lung Cancer|Pancreatic Adenocarcinoma|Pancreatic Neoplasms|Lung Cancer	DRUG: PT199|DRUG: Anti-PD-1 monoclonal antibody	PHASE1	23 June 2022
NCT05119998	Solid Tumor	DRUG: IBI325 + sintilimab|DRUG: IBI325	PHASE1	8 February 2022
NCT05246995	Solid Tumor	DRUG: IBI325 + Sintilimab	PHASE1	23 March 2022

Source of information: ClinicalTrials.gov listings. All information is accessed on 2 September 2023.

**Table 2:** Studies targeting A_2a_R or A_2b_R in cancer.

Study ID	Conditions	Interventions	Phase	Start Date
NCT04969315	Renal Cell Cancer|Castrate Resistant Prostate Cancer|Non-Small-Cell Lung Cancer	DRUG: TT-10	PHASE1|PHASE2	1 May 2023
NCT04976660	Colorectal Cancer|Gastric Cancer|Hepatocellular Carcinoma|Pancreatic Cancer	DRUG: TT-4	PHASE1|PHASE2	15 December 2022

Source of information: ClinicalTrials.gov listings. All information is accessed on 2 September 2023.

**Table 3:** Studies targeting CD38 in cancer.

Study ID	Conditions	Interventions	Phase	Start Date
NCT03319667	Plasma Cell Myeloma	DRUG: Isatuximab SAR650984|DRUG: Bortezomib|DRUG: Lenalidomide|DRUG: Dexamethasone	PHASE3	7 December 2017
NCT03275285	Plasma Cell Myeloma	DRUG: isatuximab SAR650984|DRUG: carfilzomib|DRUG: dexamethasone	PHASE3	25 October 2017
NCT05461209	Relapsed/Refractory Multiple Myeloma	DRUG: Talquetamab|DRUG: Belantamab Mafodotin	PHASE3	20 October 2022
NCT05572515	Relapsed or Refractory Multiple Myeloma	DRUG: Teclistamab|DRUG: Pomalidomide|DRUG: Bortezomib|DRUG: Dexamethasone|DRUG: Carfilzomib	PHASE3	29 March 2023
NCT04270409	Plasma Cell Myeloma	DRUG: Isatuximab SAR650984|DRUG: Lenalidomide|DRUG: Dexamethasone	PHASE3	16 June 2020
NCT02419118	Multiple Myeloma	DRUG: Daratumumab|DRUG: Lenalidomide|DRUG: Dexamethasone	PHASE2|PHASE3	15 January 2023
NCT02990338	Plasma Cell Myeloma	DRUG: Isatuximab|DRUG: Pomalidomide|DRUG: Dexamethasone	PHASE3	22 December 2016
NCT03937635	Smoldering Plasma Cell Myeloma	BIOLOGICAL: Daratumumab|DRUG: Dexamethasone|DRUG: Lenalidomide|OTHER: Quality-of-Life Assessment|OTHER: Questionnaire Administration	PHASE3	16 September 2019

Source of information: ClinicalTrials.gov listings. All information is accessed on 2 September 2023.

**Table 4:** Studies targeting multiple sites in cancer.

**Study ID**	Conditions	Interventions	Phase	Start Date
NCT04306900	Solid Tumor, Adult	COMBINATION_PRODUCT: TTX-030, budigalimab and mFOLFOX6|COMBINATION_PRODUCT: TTX-030, budigalimab and docetaxel|COMBINATION_PRODUCT: TTX-030 and mFOLFOX6|COMBINATION_PRODUCT: TTX-030 and budigalimab|COMBINATION_PRODUCT: TTX-030, budigalimab, nab-paclitaxel and gemcitabine|COMBINATION_PRODUCT: TTX-030 and pembrolizumab|COMBINATION_PRODUCT: TTX-030, nab-paclitaxel and gemcitabine|COMBINATION_PRODUCT: Budigalimab and mFOLFOX6	PHASE1	30 March 2020
NCT03884556	Solid Tumor|Lymphoma	DRUG: TTX-030|DRUG: Pembrolizumab|DRUG: Gemcitabine|DRUG: nab paclitaxel	PHASE1	10 April 2019
NCT05177770	Metastatic Castration-resistant Prostate Cancer|Prostate Cancer	DRUG: SRF617|DRUG: etrumadenant|DRUG: zimberelimab	PHASE2	17 January 2022
NCT04381832	Prostatic Neoplasms, Castration-Resistant|Androgen-Resistant Prostatic Neoplasms|Castration Resistant Prostatic Neoplasms|Prostatic Cancer, Castration-Resistant	DRUG: Etrumadenant|DRUG: Zimberelimab|DRUG: Quemliclustat|DRUG: Enzalutamide|DRUG: Docetaxel|DRUG: SG	PHASE1|PHASE2	7 July 2020
NCT05329766	Gastrointestinal Tract Malignancies	DRUG: Domvanalimab|DRUG: Quemliclustat|DRUG: Zimberelimab|DRUG: Fluorouracil|DRUG: Leucovorin|DRUG: Oxaliplatin	PHASE2	10 June 2022
NCT05688215	Borderline Resectable Pancreatic Adenocarcinoma|Locally Advanced Pancreatic Ductal Adenocarcinoma	PROCEDURE: Biospecimen Collection|PROCEDURE: Computed Tomography|PROCEDURE: Core Biopsy|DRUG: Fluorouracil|DRUG: Irinotecan|DRUG: Leucovorin|DRUG: Leucovorin Calcium|DRUG: Oxaliplatin|DRUG: Quemliclustat|DRUG: Zimberelimab	PHASE1|PHASE2	7 March 2023
NCT04660812	Metastatic Colorectal Cancer	DRUG: AB680|DRUG: Etrumadenant|DRUG: Zimberelimab|DRUG: Bevacizumab|DRUG: m-FOLFOX-6 regimen|DRUG: Regorafenib	PHASE1|PHASE2	10 May 2021
NCT04381832	Prostatic Neoplasms, Castration-Resistant|Androgen-Resistant Prostatic Neoplasms|Castration Resistant Prostatic Neoplasms|Prostatic Cancer, Castration-Resistant	DRUG: Etrumadenant|DRUG: Zimberelimab|DRUG: Quemliclustat|DRUG: Enzalutamide|DRUG: Docetaxel|DRUG: SG	PHASE1|PHASE2	7 July 2020
NCT04660812	Metastatic Colorectal Cancer	DRUG: AB680|DRUG: Etrumadenant|DRUG: Zimberelimab|DRUG: Bevacizumab|DRUG: m-FOLFOX-6 regimen|DRUG: Regorafenib	PHASE1|PHASE2	10 May 2021
NCT05177770	Metastatic Castration-resistant Prostate Cancer|Prostate Cancer	DRUG: SRF617|DRUG: etrumadenant|DRUG: zimberelimab	PHASE2	17 January 2022
NCT04381832	Prostatic Neoplasms, Castration-Resistant|Androgen-Resistant Prostatic Neoplasms|Castration Resistant Prostatic Neoplasms|Prostatic Cancer, Castration-Resistant	DRUG: Etrumadenant|DRUG: Zimberelimab|DRUG: Quemliclustat|DRUG: Enzalutamide|DRUG: Docetaxel|DRUG: SG	PHASE1|PHASE2	7 July 2020
NCT04660812	Metastatic Colorectal Cancer	DRUG: AB680|DRUG: Etrumadenant|DRUG: Zimberelimab|DRUG: Bevacizumab|DRUG: m-FOLFOX-6 regimen|DRUG: Regorafenib	PHASE1|PHASE2	10 May 2021
NCT05177770	Metastatic Castration-resistant Prostate Cancer|Prostate Cancer	DRUG: SRF617|DRUG: etrumadenant|DRUG: zimberelimab	PHASE2	17 January 2022

Source of information: ClinicalTrials.gov listings. All information is accessed on 2 September 2023.
